# MUC4 is negatively regulated through the Wnt/β-catenin pathway via the Notch effector Hath1 in colorectal cancer

**DOI:** 10.18632/genesandcancer.108

**Published:** 2016-05

**Authors:** Priya Pai, Satyanarayana Rachagani, Punita Dhawan, Yuri M. Sheinin, Muzafar A. Macha, Asif Khurshid Qazi, Seema Chugh, Moorthy P. Ponnusamy, Kavita Mallya, Ramesh Pothuraju, Surinder K. Batra

**Affiliations:** ^1^ Department of Biochemistry and Molecular Biology, University of Nebraska Medical Center (UNMC), Omaha, NE, USA; ^2^ Department of Pathology and Microbiology, UNMC, Omaha, NE, USA; ^3^ Eppley Institute for Research in Cancer and Allied Diseases, UNMC, Omaha, NE, USA; ^4^ Fred and Pamela Buffett Cancer Center, UNMC, Omaha, NE, USA

**Keywords:** MUC4, Wnt, colorectal cancer, β-catenin, Hath1

## Abstract

MUC4 is a transmembrane mucin lining the normal colonic epithelium. The aberrant/*de novo* over-expression of MUC4 is well documented in malignancies of the pancreas, ovary and breast. However, studies have reported the loss of MUC4 expression in the majority of colorectal cancers (CRCs). A *MUC4* promoter analysis showed the presence of three putative TCF/LEF sites, implying a possible regulation by the Wnt/β-catenin pathway, which has been shown to drive CRC progression. Thus, the objective of our study was to determine whether *MUC4* is regulated by β-catenin in CRC. We first knocked down (KD) β-catenin in three CRC cell lines; LS180, HCT-8 and HCT116, which resulted in increased *MUC4* transcript and MUC4 protein. Additionally, the overexpression of stabilized mutant β-catenin in LS180 and HCT-8 resulted in a decrease in MUC4 expression. Immunohistochemistry (IHC) of mouse colon tissue harboring tubular adenomas and high grade dysplasia showed dramatically reduced Muc4 in lesions relative to adjacent normal tissue, with increased cytosolic/nuclear β-catenin. Luciferase assays with the complete *MUC4* promoter construct p3778 showed increased *MUC4* promoter luciferase activity in the absence of β-catenin (KD). Mutation of all three putative TCF/LEF sites showed that *MUC4* promoter luciferase activity was increased relative to the un-mutated promoter. Interestingly, it was observed that MUC4 expressing CRC cell lines also expressed high levels of *Hath1*, a transcription factor repressed by both active Wnt/β-catenin and Notch signaling. The KD of β-catenin and/or treatment with a Notch γ-secretase inhibitor, Dibenzazepine (DBZ) resulted in increased *Hath1* and MUC4 in LS180, HCT-8 and HCT116. Furthermore, overexpression of Hath1 in HCT-8 and LS180 caused increased *MUC4* transcript and MUC4 protein. Taken together, our results indicate that the Wnt/β-catenin pathway suppresses the Notch pathway effector Hath1, resulting in reduced *MUC4* in CRC.

## INTRODUCTION

Colorectal cancer (CRC) is the third leading cause of cancer deaths in the United States, accounting for 49,700 estimated total deaths in the year 2015 alone [1]. CRC is characterized by the mutational inactivation of tumor suppressor genes such as Adenomatous polyposis coli gene (APC), p53 and components of the TGF-β pathway as well as activation of oncogenes such as *KRAS* [2]. Most frequently, tumors possess mutations in the APC gene, causing the activation of the canonical Wnt pathway [2]. A small subset of patients possess activating mutations in β-catenin, also resulting in the activation of the Wnt/β-catenin pathway [2]. The majority of CRCs (70-80%) possess APC mutations [2] and certain individuals may possess germline mutations in APC, as in familial adenomatous polyposis, where virtually all those afflicted individuals develop CRC by age 40 [3].

Precursor lesions typically follow a polyp-adenoma- carcinoma sequence. The degree of nuclear β-catenin progressively increases during CRC progression, as a consequence of mutations in APC/β-catenin [3]. Another feature associated with early CRC progression is the presence of dysplastic crypts or aberrant crypt foci (ACF) [4]. These lesions precede the formation of adenomas and are associated with mucin depleted foci (MDF) [4, 5]. MDFs are characterized by the absence of mucins and were originally identified in the colon of rats treated with the carcinogens Azoxymethane (AOM) and dextran sodium sulfate (DSS) [4, 5].

Mucins are high molecular weight glycoproteins and usually line the epithelial surfaces of the digestive and reproductive tracts [6]. MUC4 ordinarily lines the goblet cells and epithelial cells of the normal human small and large intestine [6]. A number of studies have suggested that MUC4 expression is generally lost in CRC [7, 8]. However, certain other studies suggest that while the majority (around 75%) of CRC tumors have reduced or zero MUC4 expression relative to normal tissue, the subset (around 25%) that have high MUC4 expression have a worse prognosis, specifically in the early stages (stage I and II) of the disease [9, 10]. Furthermore, data extracted from the Oncomine database (www.oncomine.org) shows that *MUC4* is among the top 5% most significantly downregulated genes in colorectal cancers compared to normal tissue as per 3 studies (p value < 1E-4). A recent study from our lab corroborated these findings *via* the immunohistochemistry of premalignant and malignant tissues, which showed MUC4 was significantly reduced in the adenoma-carcinoma sequence relative to normal tissue [11]. Thus, while the precise role played by MUC4 in CRC progression is unclear, most studies indicate that MUC4 expression is lost in CRC.

A number of studies have probed the effect of perturbations in the Wnt pathway on mucins in CRC. When a siRNA targeting β-catenin was used in the CRC cell line LS174, increased general mucin production (measured by Periodic-acid/Schiff (PAS) staining) was observed [12]. The most abundantly expressed mucin in the normal colon, MUC2, is repressed by β-catenin via an indirect mechanism involving Sox9 in CRC [13]. The Wnt/β-catenin pathway also indirectly regulates the level of mucins in CRC, via regulation of the Notch target, Hath1 [14]. Hath1 (Atoh1) is suppressed by the Notch signaling target Hes1 [15]. Hath1 is a basic helix-loop-helix transcription factor that binds E box motifs in the promoter elements of target genes [16]. In the colon, active Hath1 driven transcription governs the secretory fate of colonocytes [17]. Both the Notch and Wnt pathways have been shown to be synergistic in CRC progression, in part, by the suppression of Hath1 [15]. Hath1 is a tumor suppressor in CRC and its expression is reduced in the majority of CRCs [14]. The Wnt/β-catenin pathway has been shown to directly reduce Hath1 at the protein as well as the RNA level in CRC [14, 18]. Hath1 has also been shown to regulate MUC2 in CRC [14] and MUC5AC and MUC6 in gastric cancer [19]. Importantly, a *MUC4* promoter analysis in the present study showed the presence of a putative Hath1 binding site at −3102/−3089. Thus, a number of factors collude to alter the expression of mucins in CRC.

The *MUC4* promoter has been well characterized and is approximately 3.7 kilobases in size; a TATA box is present at −2672/−2668 upstream of ATG [20], dividing it into proximal and distal promoter regions. A *MUC4* promoter analysis revealed the presence of three putative TCF/LEF sites; one in the proximal promoter at −2612 (Site #1), and two in the distal promoter at positions −3226 (Site #2) and −3408 (Site #3). In summation, studies thus far show that β-catenin KD increases mucin production in CRC. In addition, the *MUC4* promoter was found to possess 3 putative TCF/LEF sites. We thus hypothesized that Wnt/β-catenin can repress *MUC4* in CRC. In addition, the Wnt/β-catenin pathway has been shown to repress the transcription factor Hath1, which also governs expression of mucins such as MUC2 and MUC5AC. Notably, the *MUC4* promoter also contains a putative Hath1 site. Thus, we further hypothesized that the β-catenin mediated repression of *MUC4* may occur via the repression of Hath1, which ordinarily upregulates MUC4 in CRC.

## RESULTS

### The loss of MUC4 corresponds with an increasing degree of nuclear β-catenin in CRC

**Figure 1:**
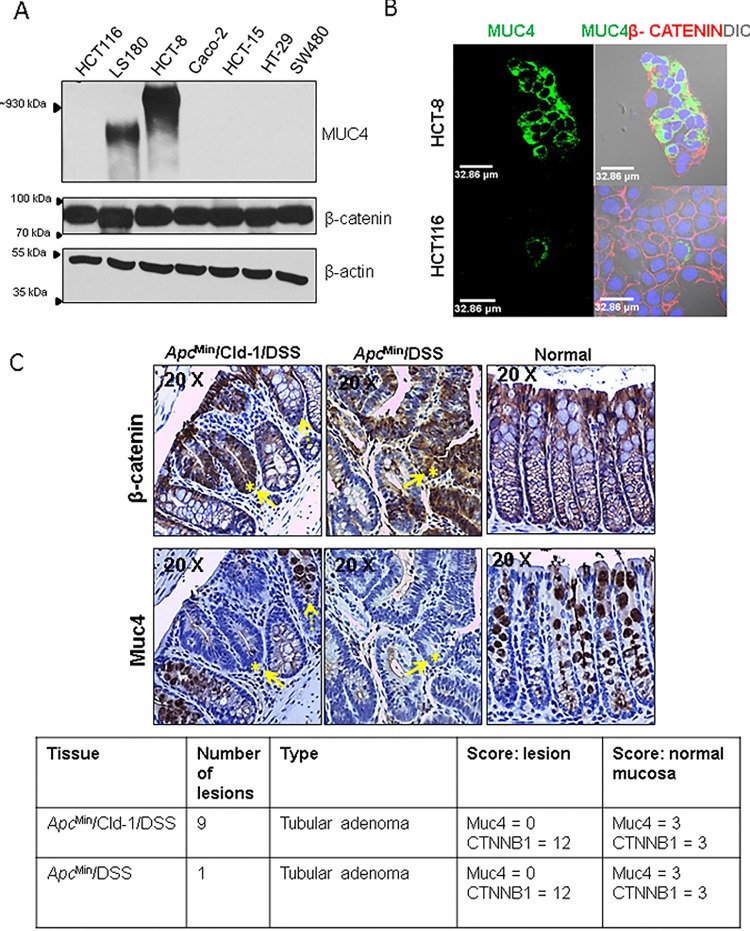
Increased nuclear β-catenin is associated with reduced MUC4 expression (A) A panel of CRC cell lines was profiled for the expression of MUC4 and β-catenin. β-actin was used as a loading control. (B) Confocal microscopy with MUC4 (green) antibody shows that HCT-8 cells express MUC4 abundantly while HCT116 cells show very low MUC4 expression. β-catenin (red) is present in both cell lines.(C) Immunohistochemical staining for mouse Muc4 and β-catenin in colon sections from *Apc*^Min^/Cldn-1 mice treated with DSS (left panel), *Apc*^Min^ mice treated with DSS (middle) and *Apc*^Min^ given water (right panel). Staining for β-catenin (upper panel) and Muc4 (lower panel) showed intense cytosolic/nuclear staining for β-catenin and depletion of Muc4 in lesions (solid arrow), while surrounding normal areas showed reduced β-catenin and intense goblet cell staining for Muc4 (dotted arrow). Table shows type, number of lesions in in mice either treated with DSS alone or *Apc*^Min^ mice treated with DSS.

In order to determine whether aberrant β-catenin expression/localization correlated with MUC4 expression in CRC, we first examined the expression of MUC4 and β-catenin in CRC cell lines. While all cell lines examined expressed β-catenin, only two of the 7 cell lines were found to express MUC4 abundantly; LS180 and HCT-8 (Figure 1A), while MUC4 expression in HCT116 was negligible to none. However, with confocal microscopy, HCT116 was observed to express low levels of MUC4, in contrast with the HCT-8 cell line, where MUC4 was abundantly expressed (Figure 1B). Thus, a majority of the CRC cell lines examined displayed a loss of MUC4.

In order to determine whether the expression of MUC4 is correlated with β-catenin *in vivo*, we examined Muc4 and β-catenin levels in colon tissue sections procured from three groups of mice: *Apc*^Min^ mice that were treated with DSS (*Apc*^Min^/DSS), *Apc*^Min^ mice overexpressing Claudin-1, which represents a more aggressive model of colorectal tumorigenesis, also treated with DSS (*Apc*^Min^/Cldn-1/ DSS) and lastly, *Apc*^Min^ mice that were given water, representing a control group. Colon tumorigenesis can be induced in *Apc*^Min^ mice by administration of the colitis inducing DSS [21]. An earlier study showed that the overexpression of Claudin-1 led to accelerated colorectal tumorigenesis [22], thus sections from these mice were also examined in addition to *Apc*^Min^/DSS sections alone. As expected, the section from the *Apc*^Min^/Cldn-1 mouse treated with DSS possessed 9 adenomatous polyps and displayed an increased cytosolic/nuclear β-catenin in the lesions in comparison to the adjacent normal regions. These lesions also displayed a virtual absence of Muc4, while adjacent normal regions showed intense Muc4 staining, particularly in goblet cells (Figure 1C). The goblet cells in normal areas showed strong cytoplasmic staining, while lesions showed occasional faint apical staining. Similar results were obtained with the *Apc*^Min^/DSS tissue section, which possessed 1 tubular adenoma (Figure 1C). In contrast, sections from *Apc*^Min^ mice treated with water showed normal histology with strong cytoplasmic staining of Muc4 in goblet cells. In addition, tissue from a CDX2P- NLS-Cre; Apc^loxP/+^ mouse showed an increased nuclear β-catenin concomitant with markedly reduced Muc4 in high grade dysplastic lesions (Supplementary Figure 1A). Thus, lesions in the mouse colon showed a marked loss of Muc4 expression along with intense cytosolic/nuclear β-catenin.

In concordance with results from earlier studies [10, 23], it was observed that the expression of MUC4 was lower in human polyp sections in comparison to the normal colon. The polyp section examined showed a loss of MUC4 expression, concurrent with an increased expression (cytoplasmic/nuclear) of β-catenin relative to the normal colon section (Supplementary Figure 1B).

### Knock down of β-catenin induces the expression of MUC4 in CRC

**Figure 2:**
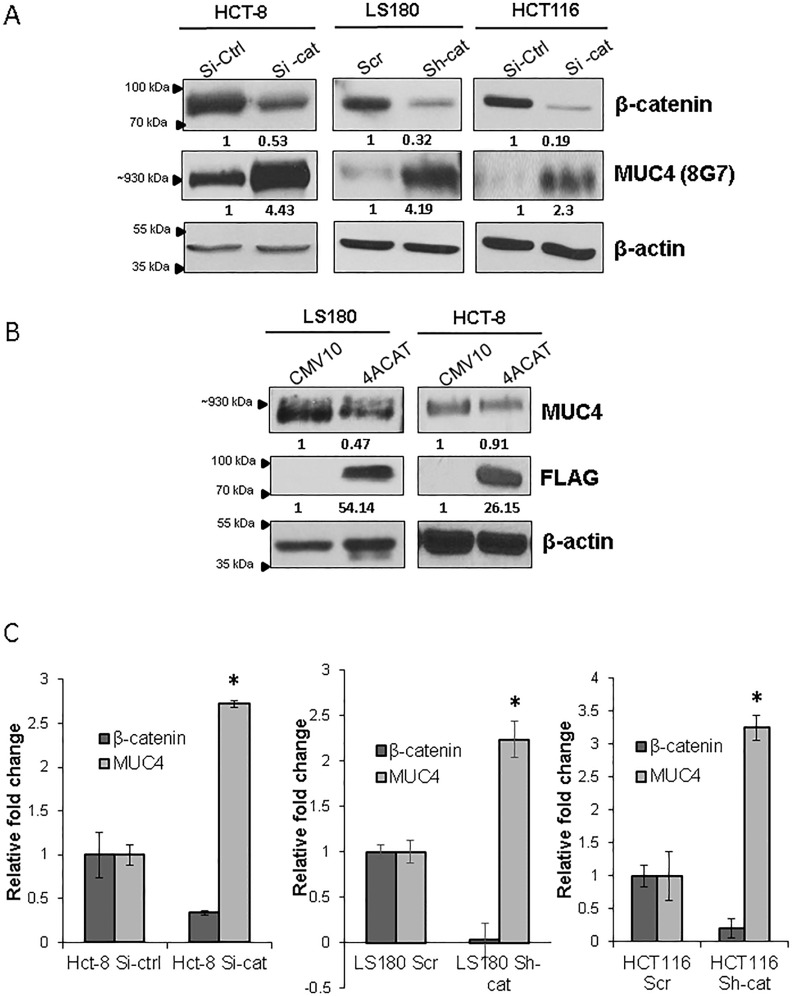
Knockdown (KD) of β-catenin induces MUC4 expression (A) Lentiviral shRNA and siRNA were used to KD β-catenin in HCT-8, HCT116, and LS180. The levels of MUC4 protein were increased in the KD cells when probed with the 8G7 MUC4 antibody. (B) A FLAG tagged stabilized β-catenin construct (4ACAT) was transiently transfected in LS180 and HCT-8. The levels of MUC4 protein were reduced. (C) Real time PCR was used to assess *MUC4* mRNA levels upon β-catenin KD. (* p< 0.05).

In order to delineate the precise relationship between MUC4 and β-catenin in CRC, we knocked down (KD) β-catenin using lentiviral shRNA as well as siRNA in three CRC cell lines: HCT-8, HCT116 and LS180. Interestingly, we were unable to establish a stable β-catenin KD in all of CRC cell lines, likely due to the reliance of these cells upon β-catenin for survival. Upon the KD of β-catenin, there was an increase in MUC4 in all three cell lines (Figure 2A). Since the 8G7 antibody used to detect MUC4 protein targets the variable number of tandem repeats (VNTR) domain and may therefore be affected by variations in the glycosylation of the protein, we used the 2214 antibody (targeting the MUC4-α-N-Ter [24]) to confirm that the increase in MUC4 upon KD of β-catenin was due to increase in actual protein levels as opposed to altered glycosylation leading to enhanced detection by the 8G7 antibody (Supplementary Figure 2).

**Figure 3:**
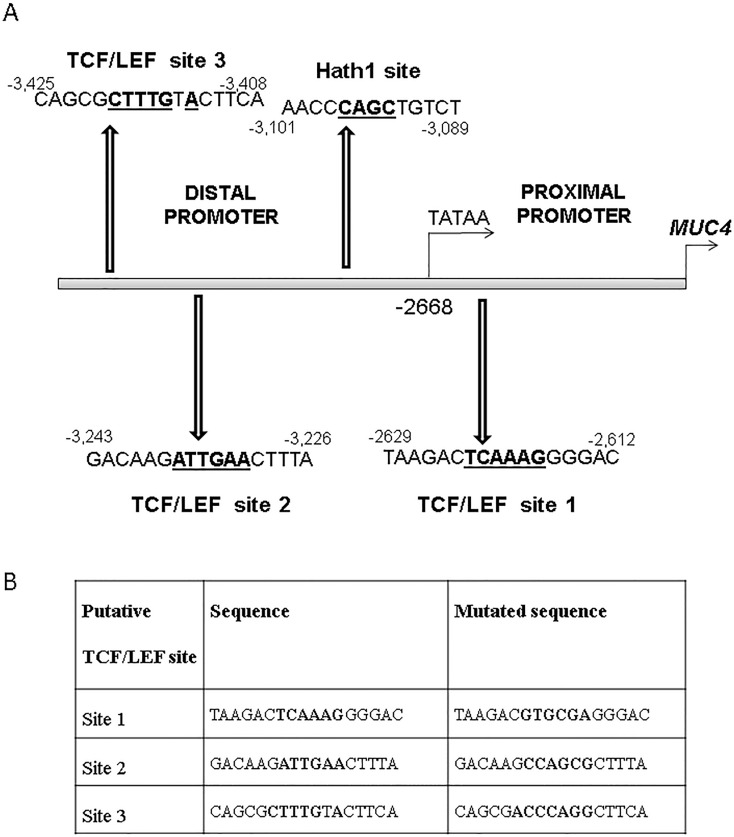
Analysis of the MUC4 promoter (A) *MUC4* promoter analysis showed the presence of three putative TCF/LEF sites and one Hath1 site. Positions of each putative site relative to ATG are indicated, as are the sequences of each of the sites, with the core sequence in bold letters. (B) Site-directed mutagenesis was used to mutate each of the three TCF/LEF sites to the sequence indicated.

For further confirmation of the above findings, we transiently overexpressed a stabilized β-catenin construct, 4ACAT, which resulted in decreased MUC4 expression in the LS180 and HCT-8 cell lines (Figure 2B). In order to determine whether the β-catenin KD induced MUC4 up- regulation occurred at the transcript level, we examined the *MUC4* transcript levels in all three cell lines and we confirmed that the *MUC4* RNA was also increased upon β-catenin KD (Figure 2C). In order to determine whether *MUC4* was repressed at the transcript level, we performed a *MUC4* promoter analysis, which showed the presence of three putative TCF/LEF sites as well as one putative Hath1 site (Figure 3A). A *MUC4* promoter luciferase construct was generated, p3778, encompassing the entire promoter cloned into the pGL4.17 vector and each of the three putative TCF/LEF sites were mutated individually, as enumerated in Figure 3B.

### Luciferase studies with *MUC4* promoter construct show that MUC4 can be governed by β-catenin

**Figure 4:**
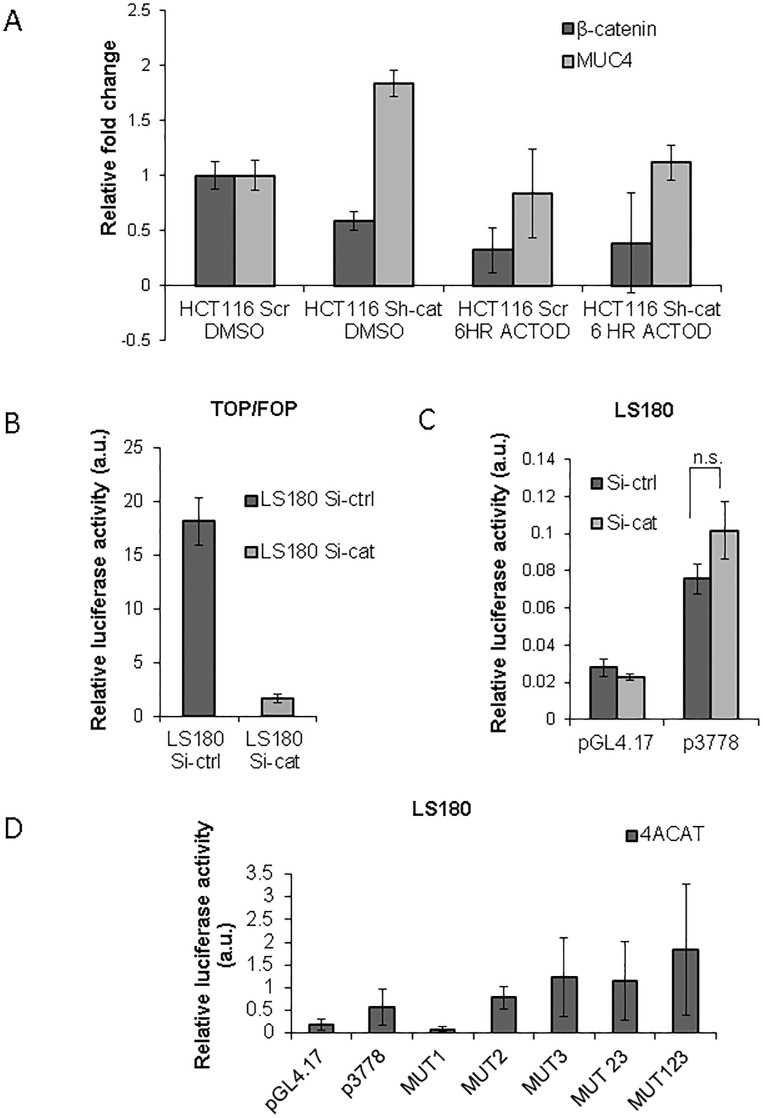
The Wnt/ β-catenin pathway represses MUC4 transcription (A) β-catenin does not increase *MUC4* transcript stability. HCT116 Scr and HCT116 Sh-cat were treated with 10μg/ml Actinomycin D. After 6 hours of treatment, the levels of MUC4 mRNA were not enriched in the Sh-cat cells in comparison to the Scr Actinomycin D treated cells. (B) Luciferase studies with the *MUC4* promoter luciferase construct. LS180 cells were transiently transfected with si-RNA targeting β-catenin. TOP/FOPflash studies showed that there was reduced β-catenin mediated transcription in the cells transfected with siRNA. (C) When siRNA transfected cells were also transfected with the p3778 MUC4 promoter luciferase construct, there was an increase in *MUC4* promoter driven luciferase activity. (D) The p3778 promoter construct with each of the three putative TCF/LEF sites mutated (*i.e.,* −2612:MUT1, −3226:MUT2, −3408: MUT3) was transfected into LS180 cells in the presence of 4ACAT. The pCMV9-Renilla vector was used as an internal transfection control; all luciferase experiments were performed in triplicate and repeated three times. Images represent the average of at least three experiments, each performed in triplicate.

In light of the observation that *MUC4* was increased upon β-catenin KD, we decided to ascertain whether β-catenin can affect *MUC4* RNA stability. For this purpose, we treated our HCT116 Scr and Sh-cat cells with Actinomycin D (10μg/ml) for 6 hours. It was observed that there was a reduction in *MUC4* (the half-life of *MUC4* mRNA is 5 hours [25]), concurrent with the reduction in *β-catenin* in both the HCT116 Scr and sh-cat cells (Figure 4A), thus indicating that β-catenin KD does not increase the mRNA stability of *MUC4*.

In order to determine whether *MUC4* transcript levels can be directly altered by β-catenin, we used the *MUC4* promoter luciferase construct, p3778, which encompasses all three putative TCF/LEF sites. LS180 cells were transfected with a β-catenin si-RNA as well as the TOPflash plasmid and its negative control FOPflash, which are a measure of the β-catenin/TCF signaling. As expected, there was a decrease in the TOP/FOPflash luciferase activity in LS180 β-catenin siRNA transfected cells in comparison to the control (Si-ctrl) cells (Figure 4B). We then transfected the LS180 Si-cat and Si-ctrl cells with the *MUC4* promoter luciferase construct p3778. There was an increased *MUC4* promoter driven luciferase activity in the Si-cat cells in comparison to the Si-ctrl transfected cells (Figure 4C). However, this difference was not statistically significant.

Since our promoter analysis indicated the presence of three putative TCF/LEF sites in the *MUC4* promoter: at positions −2612, −3226 and −3408, i.e., site #1, site #2 and site #3 respectively, we mutated the TCF/LEF sites both individually as well as in combination (MUT1, MUT2, MUT3, and MUT123) in the p3778 construct. It was observed that while transfection with MUT1 caused a reduction in the luciferase activity (Figure 4D), MUT2, MUT3 and MUT123 caused an increase in luciferase activity relative to the un-mutated promoter in the presence of 4ACAT, suggesting that either site 2 or 3 or all 3 TCF/LEF sites represses *MUC4*. Since MUT1 decreased luciferase activity and therefore appeared to promote *MUC4* transcription, we generated another construct MUT23, which possessed an intact site #1 but mutant site #2 and #3. This luciferase reading was higher than that of p3778 but lower than MUT123, implying that mutant site #1 also contributes to increased *MUC4* transcription. Although mutation of all three TCF/LEF sites resulted in an increase in *MUC4* promoter driven luciferase activity, overall, this difference was not statistically significant.

### MUC4 expression is also regulated by Hath1

**Figure 5:**
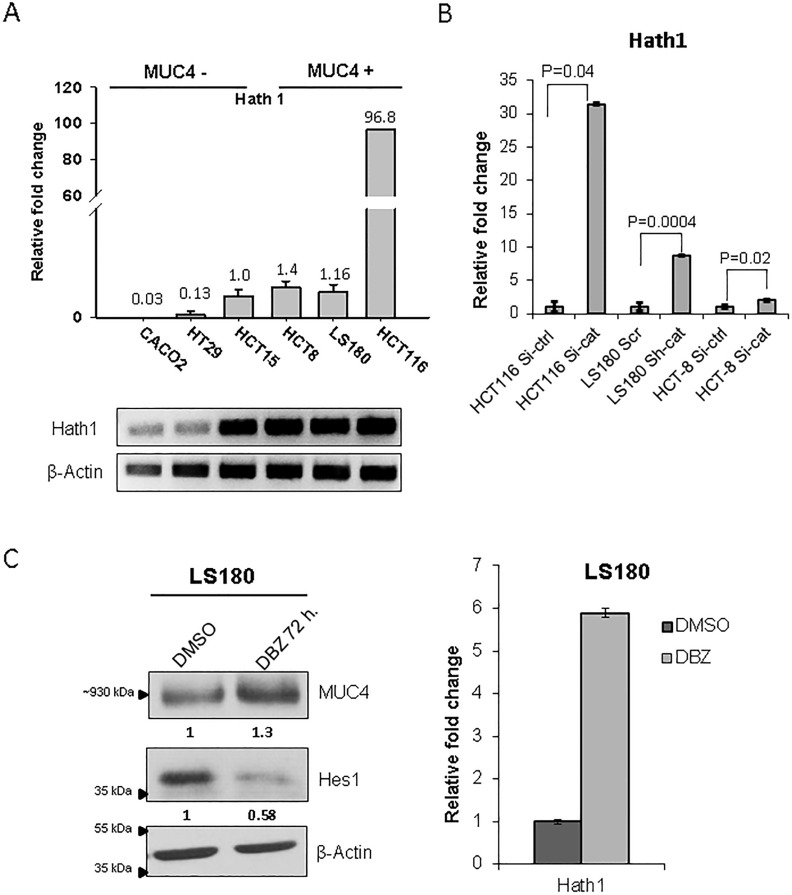
Hath1 expression is associated with increased MUC4 (A) Real time PCR for Hath1 was performed in a panel of cell lines. The PCR products were then run on a 2% agarose gel. β-actin was used as a reference gene. (B) Real time PCR for Hath1 in β-catenin KD CRC cells showed a significant increase in *Hath1* levels. P values are as indicated. (C) LS180 cells were treated with the γ secretase inhibitor DBZ (500nm), which resulted in an increase in MUC4. Hes1, a Notch pathway target gene, was used as a verification of treatment efficacy.

Although luciferase studies with the *MUC4* promoter constructs indicated that the *MUC4* transcript can be governed by β-catenin, the results were not statistically significant. Therefore, it was decided to explore the possibility that β-catenin governs MUC4 expression *via* an indirect mechanism. The Wnt/β-catenin pathway has been shown to repress Hath1 expression [14], which in turn, regulates the expression of mucins such as MUC5AC and MUC2 [19]. We therefore examined the level of *Hath1* in CRC cell lines. Interestingly, it was seen that the *Hath1* was higher in MUC4 expressing cell lines, HCT116, HCT8, and LS180 in comparison to MUC4 non- expressing cell lines HCT-15, Caco-2 and HT-29 (Figure 5A). Furthermore, we observed that there was a significant increase in *Hath1* mRNA expression upon β-catenin KD (Figure 5B). In order to modulate the levels of Hath1 in CRC cell lines, we treated LS180 with 500nM DBZ, a γ-secretase inhibitor, which has been shown to increase Hath1 [26] for 72 hours. There was an increase in MUC4 (Figure 5C) in the DBZ treated cells in comparison to the DMSO treated control cells, concurrent with an increase in *Hath1,* suggesting a Hath1-MUC4 regulatory relationship.

**Figure 6:**
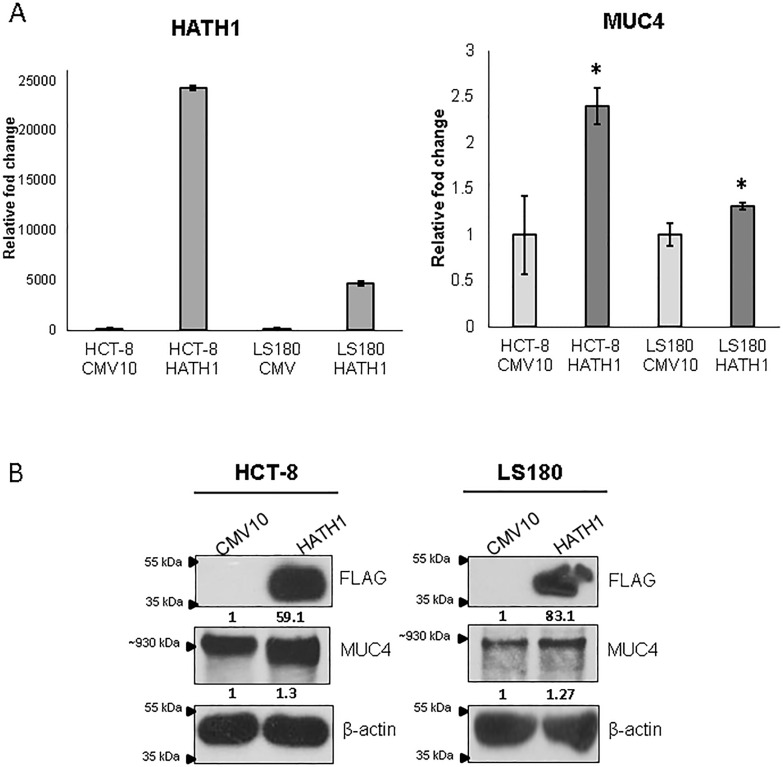
Overexpression of Hath1 results in increased MUC4 (A) HCT-8 cells were transfected with a Hath1 overexpression construct. Real time PCR was used to confirm the overexpression of Hath1 as well as *MUC4* RNA levels. It was observed that *MUC4* was increased in Hath1 transfected cells in comparison to cells transfected with the empty vector. (* p< 0.05) (B) Western blot analysis shows that the overexpression of FLAG-tagged Hath1 results in increased MUC4 protein in HCT-8.

For further confirmation of a Hath1-MUC4 regulatory relationship, a Hath1 over-expression construct was generated. When transiently over-expressed in the HCT-8 and LS180 cell lines, an increase in *MUC4* RNA and protein was observed (Figure 6A and B).

## DISCUSSION

Aberrations in the Wnt/β-catenin pathway are well established initiating events in CRC [2, 3, 27]. Most frequently, truncating mutations in APC, present in 85-90% of all tumors, prevents the phosphorylation mediated degradation of β-catenin and drives the molecule into the nucleus [2, 3]. Less frequently, activating mutations in β-catenin that prevent its degradation and mutations in Axin2/1 can also cause activation of the Wnt/β-catenin pathway [2, 3]. Notably, aberrant activation of the Wnt/ β-catenin pathway is the only abnormality seen in early CRC precursor lesions such as aberrant crypt foci and adenomas [28]. The Wnt/β-catenin pathway activates the transcription of a host of tissue specific genes [29]. In the colon, β-catenin causes the loss of differentiation of CRC cells and pushes the cells into a crypt progenitor phenotype, typically associated with a general loss of mucin expression [28]. This includes the loss of mucin expression, chiefly MUC2, and the gain of several genes commonly active in the proliferative base of the normal colonic crypt such as CD44 [28].

As hinted at previously, the loss of mucin expression is one of the defining characteristics of precursor lesions such as aberrant crypt foci and mucin depleted foci. The most extensively studied mucin in CRC is MUC2, which is the main secreted mucin in the colon [30]. MUC2 expression is usually lost during CRC progression and this loss has been shown to be mediated by β-catenin, albeit *via* an indirect mechanism involving SOX9 [13]. SOX9 is a transcription factor that is expressed in the proliferative compartment of intestinal crypts and its expression coincides with regions harboring active Wnt signaling [13]. Wnt/β-catenin signaling was found to regulate SOX9, which in turn was found to directly repress markers of differentiation such as *MUC2* and *CDX2* [13]. Other factors, such as the loss of Hath1, a transcription factor that ordinarily governs the secretory fate of colonocytes, contribute to the loss of MUC2 expression [14]. Overexpression of Hath1 led to an increase in MUC2 levels. Conversely, the inhibition of Wnt/β-catenin led to an increase in MUC2. Hath1 has also been shown to be repressed by active Wnt/β-catenin signaling [18]. The loss of MUC2 expression has also been shown to aid CRC progression. The loss of Muc2 alone was found to result in CRC in a mouse model [31]. Furthermore, the Wnt/β-catenin pathway and Muc2 loss were found to cooperatively accelerate CRC progression [32]. Thus, the loss of MUC2, which is likely mediated in part by the Wnt/β-catenin pathway via two disparate mechanisms, typically occurs during the polyp-adenoma-carcinoma sequence and contributes to CRC progression.

MUC4 is a transmembrane mucin that ordinarily lines the epithelial surface of the gastrointestinal, respiratory and reproductive tracts [6]. In the human colon, MUC4 is typically expressed in goblet cells and in the lower two-thirds of the normal crypt [23], while its expression in CRC progression has been the subject of some controversy. Although most studies concur that the majority of CRCs display a loss/reduced MUC4 expression, somewhat conflictingly, it has been proposed that MUC4 expression, when present, confers a worse prognosis to patients with early stage (grade I and II) CRCs [10, 33]. A meta-analysis of all patient data showed that MUC4 expression was associated with a poorer prognosis in CRC [9]. Also, a recent study from our lab showed that Muc4 expression in mice led to increased susceptibility to AOM/DSS induced colitis and CRC [34]. In one study, serrated adenomas displayed a complete loss of MUC4 expression while 50% of hyperplastic polyps showed reduced MUC4 expression and traditional adenomas (flat or sessile adenomas and polypoid adenomas) showed no change in MUC4 expression compared to normal [23]. Thus, most studies concur that MUC4 expression is reduced/lost in most CRC precursor lesions and full blown CRCs.

The current study aimed to determine whether MUC4 expression is governed by β-catenin, since the *MUC4* promoter was found to contain 3 TCF/LEF sites [35]. We first analyzed the expression pattern of MUC4 and β-catenin in seven commonly used CRC cell lines. It was observed that only two cell lines expressed MUC4 abundantly, LS180 and HCT-8. These cell lines are moderate/well differentiated and secrete Carcinoembryonic antigen (CEA) which is associated with a more differentiated and less tumorigenic state [36]. HCT116 expressed very low levels of MUC4. All three MUC4 expressing cell lines have a wild type p53 [37, 38]. The MUC4 non-expressing cell lines we examined (HCT-15, HT-29, SW480 and CaCo-2) possess a mutant p53 [37-39]. Interestingly, p53 loss typically occurs at a later stage of CRC progression [2] and therefore the MUC4 expressing cell lines may represent an earlier stage in CRC progression. Thus, a majority of the CRC cell lines examined did not express MUC4. Furthermore, IHC staining of mouse tubular adenomas suggested that MUC4 loss is associated with increased cytosolic/nuclear β-catenin.

The KD of β-catenin in the three cell lines that express MUC4 showed that there was a significant increase in MUC4 protein expression upon KD of β-catenin. This was consistent at the RNA level, where *MUC4* levels were found to be significantly higher in the KD cells. These results imply that β-catenin ordinarily represses *MUC4*; seemingly contradicting our earlier findings in pancreatic cancer, where we showed that *MUC4* is increased by β-catenin [35]. However, one must note that these two diseases are completely different entities with distinct mutational profiles and β-catenin typically has different tissue specific target genes. Moreover, nuclear β-catenin is typically 5-20 times higher in CRC than in PDAC [40], thus possibly altering levels of a different set of target genes, which, in turn, could affect factors such as *MUC4* promoter methylation and histone acetylation. Furthermore, it is possible that β-catenin acts as a molecular toggle, with high levels (as seen in CRC) repressing *MUC4* and low to moderate levels (as seen in pancreatic cancer) promoting *MUC4* transcription via a differential recruitment of co-factors. One study has shown that the *MUC4* promoter is methylated at certain key residues in the proximal promoter in the cell line Caco-2 and that treatment with the histone deacetylase inhibitor Trichostatin A and DNA methylation inhibitor 5-aza-2′-deoxycytidine caused increased *MUC4* mRNA [41].

The Wnt/β-catenin pathway has been shown to perturb the levels of numerous miRNAs in CRC [42], likely affecting mRNA levels of many genes. Moreover, *MUC4* has been shown to be targeted by several miRNAs [43, 44]. In light of these facts, we asked whether β-catenin can alter the *MUC4* mRNA. From our Actinomycin D experiment, we concluded β-catenin KD does not increase *MUC4* mRNA stability.

Having confirmed that the β-catenin KD induces increased *MUC4* mRNA and protein levels, we decided to determine whether this β-catenin induced MUC4 repression occurs via a direct or an indirect mechanism. For this, we used a *MUC4* promoter luciferase construct, p3778, which encompasses all three of the putative TCF/ LEF sites. It was observed that when LS180 cells were transfected with p3778 in the presence of β-catenin siRNA, there was an increase in the *MUC4* promoter driven luciferase activity. However, this increase was not statistically significant. Mutational studies showed that site #2 and site #3 are repressive but site #1 may promote *MUC4* transcription in the presence of 4ACAT. However, it is possible that binding of β-catenin does not occur at site #1 in CRC due to factors such as differential promoter accessibility. Despite the observed differences in *MUC4* promoter luciferase activity due to mutations in TCF/LEF sites, the differences were statistically insignificant. Future studies could examine whether β-catenin/TCF forms a repressive complex on the MUC4 promoter via chromatin immunoprecipitation (ChIP).

Owing to the fact that our promoter luciferase assays did not yield conclusive results, we went on to examine alternate pathways downstream of β-catenin that may regulate *MUC4*. We focused on Hath1 because this well-established tumor suppressor gene in CRC has been shown to be repressed by β-catenin and regulate MUC2 in CRC [14]. In concordance with earlier studies [15, 18], it was observed that β-catenin appeared to repress Hath1 in CRC. Hath1 levels increased upon the KD of β-catenin in all three cell lines examined. Furthermore, the over- expression of Hath1 resulted in increased *MUC4* RNA and protein in the HCT-8 cell line, suggesting that Hath1 may directly regulate MUC4 expression in CRC. Future studies could determine whether Hath1 binds the *MUC4* promoter via ChIP.

**Figure 7:**
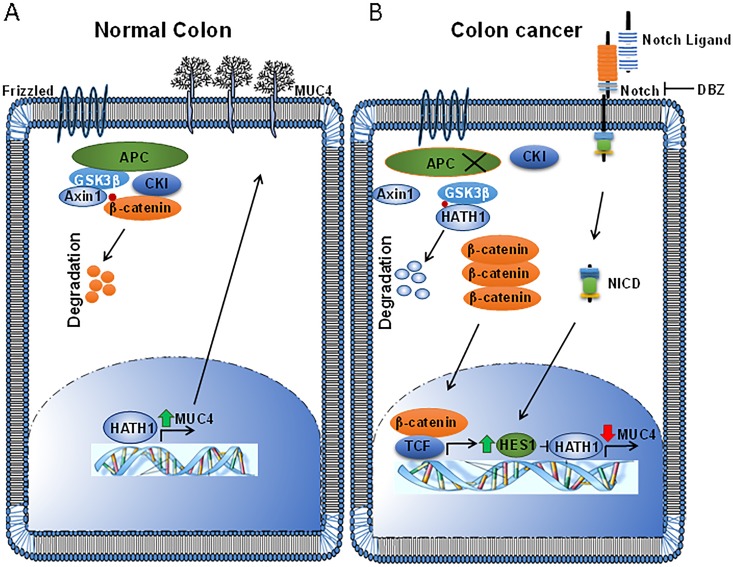
A schematic representation of the findings of this study (A) In the normal colon, Wnt signaling is inactive and Hath1 is transcriptionally active in differentiated secretory cells. Hath1 transcriptionally activates *MUC4*, as well as other mucins such as *MUC2*. (B) However, in colorectal cancer, active Wnt/β-catenin and Notch signaling transcriptionally upregulate *Hes1*. Hes1 antagonizes Hath1, thus reducing Hath1 levels. In addition, the Hath1 protein is targeted for phosphorylation mediated destruction by GSK3β instead of β-catenin, further reducing Hath1 levels. Thus, *MUC4* is no longer actively transcribed owing to reduced Hath1, resulting in reduced MUC4.

In summation, our results suggest that Hath1 may regulate *MUC4* and that during the course of CRC progression, both the Notch and Wnt pathways converge to repress Hath1 [15, 18]. This results in decreased *MUC4* transcription. The conclusions from this study have been summarized in Figure 7. In the normal differentiated secretory colonocyte (Figure 7A), Hath1 activates *MUC4* while the Wnt/β-catenin pathway is inactive. In CRC (Figure 7B), β-catenin enters the nucleus and up-regulates Hes1 [15], while the Notch pathway also activates Hes1, which antagonizes Hath1 [26, 45]. Meanwhile, Hath1 is also targeted for destruction by GSK3β mediated phosphorylation [18]. Thus, Hath1 mediated MUC4 up- regulation is abrogated, resulting in reduced MUC4. In conclusion, this study shows for the first time that Wnt/β- catenin can repress *MUC4* in CRC via the repression of the Notch effector Hath1, which ordinarily governs MUC4 in the normal colonocyte.

## MATERIALS AND METHODS

### Cell Culture

The human colorectal cancer cell lines Caco-2, HT-29, SW480, HCT116, HCT-15, HCT-8 and LS180 were purchased from American Type Culture Collection (ATCC) and cultured in α-MEM containing 10% fetal bovine serum supplemented with 100 μg/ml penicillin and streptomycin at 37°C with 5% CO_2_ in a humidified atmosphere. Cells transfected with lentiviral constructs were maintained in 5 μg/ml Puromycin as a selection agent.

### Transfection

All transient transfections were performed with Lipofectamine 2000 (Life Technologies; Carlsbad, CA, USA), as per the manufacturer's directions. For lentiviral transfection, 2×10^6^ Lenti-X-293T cell line #632180 (Clontech; Mountain View, CA, USA) cells were seeded in 10 cm dishes, and transfection was performed using a calcium phosphate precipitation method (20 μg transfer vector, 15 μg packaging plasmid, and 6 μg envelope plasmid) on the following day using the pLKO.1.sh. beta-catenin.1248 (Addgene plasmid #19761), pLKO.1 shSCR (Addgene plasmid #17920), packaging plasmid pCMV-dR8.2 dvpr and envelope plasmid pCMV-VSVG, which were a kind gift from Dr. Yuzuru Shiio (University of Tennessee Health Science Center). Supernatant was collected after 48 hours, and was concentrated using the Lenti-X™ Concentrators #631231 and #631232 from Clontech per manufacturer's instructions. After concentration, the lentiviral supernatant was used to infect 2×10^5^ target cells seeded per well of a 12-well plate. Puromycin (5 μg/ml) was used to select for positive clones. The β-catenin siRNA was purchased from Thermo Fisher Scientific, Waltham, MA USA (# 4390824, s438). For transfections, 4×10^5^ cells were seeded per well in a six well plate. The following day, 125 pmoles of the β-catenin siRNA and 125 pmoles of Silencer® Negative Control siRNA (Thermo Fisher, # AM4635) were transfected using Lipofectamine 2000 (Life Technologies; Carlsbad, CA, USA), per the manufacturer's directions, in serum free media. Fresh serum containing medium was added 4 hours following transfection. Cell lysates were made and RNA was extracted 72 hours after transfection.

### Tissues specimens, immunohistochemistry and immunofluorescence

Tissues from *Apc*^Min^ mice treated with dextran sodium sulfate (DSS) tissues and *Apc*^Min^/Cld-1/DSS mice (*Apc*^Min^-Villin-Claudin-1; generated from *Apc*^Min^ mice crossed with Villin-Claudin-1 transgenic mice) were generated as described previously [22]. The CDX2P-NLS- Cre; Apc^loxP/+^ mice, where CDX2P-NLS-Cre confers colon preferential expression of transgenes, were characterized and generated as described previously [46]. The CDX2P-NLS-Cre mice express a nuclear localized Cre recombinase regulated by a CDX2 promoter and are on a C57BL/6J genetic background. This promoter is expressed in the ileum, caecum and colon [46]. We obtained the B6.Cg-Tg(CDX2-cre)101Erf/J mice from the Jackson Laboratory (Stock No: 009350). The B6.Cg-Apc^tm2Rak^/Nci (strain number: 01XAA) mice were obtained from the National Cancer Institute (NCI) mouse repository. These mice have the exon 14 of the Apc gene flanked by loxP sites. When crossed with mice expressing a tissue specific Cre-recombinase, the loxP sites are excised by Cre- recombinase resulting in truncated Apc protein, which is 605 amino acids long, of which only the first 580 are present in the normal protein. These mice also had a C57BL/6J genetic background. Animals were maintained in accordance with guidelines and protocols approved by the Institutional Animal Care and Use Committees (IACUC) of the University of Nebraska Medical Center. The animals were exposed to a 12 hour light/dark cycle and were allowed access to food and water ad libitum. The tails of mice were clipped at the age of 8 days and the DNA was isolated using the Maxwell 16 mouse tail DNA purification kit, Promega, Madison, WI, USA. Following DNA isolation, genotyping was performed using primers listed in Supplementary Table B. Tissues were evaluated by a UNMC pathologist, Dr. Yuri Sheinin and composite score for immunohistochemical staining in mouse tissue was calculated by estimating the number of positively stained cells per hundred cells (range 1 – 4; 0 – 25 cells per hundred cells = score of 1, 26 – 50 cells per hundred cells = score 2, 51 – 75 cells per hundred cells = score 3, and 76 – 100 cells per hundred cells = score 4) and multiplying this number by the intensity of staining, which was given a range from 1-3. Pictures were taken using a Nikon Eclipse E400 light microscope (Kawasaki, Japan). The protocol for immunohistochemistry (IHC) was adapted from Kaur *et al* [47] with the following modifications: 5% hydrogen peroxide in methanol was used to block endogenous peroxide activity and the duration of this treatment was extended to 1.5 hours instead of 1 hour. Tissue immunofluorescence was performed as described previously [48]. Images were taken using an LSM 710 Zeiss Confocal Microscope located at the UNMC Advanced Microscopy Core Facility.

### Luciferase Assays

For the luciferase assays, 2×10^5^ cells were seeded in triplicate per well of 12 well plate and were transfected the next day. The pGL4.17 vector was gifted by Dr. Robert Bennett, UNMC. The M50 Super 8x TOPFlash contains seven TCF/LEF-binding sites upstream of firefly luciferase, while the negative control M51 Super 8x FOPFlash plasmid contains seven mutant TCF/LEF sites (Addgene plasmids # 12456 and # 12457, both were gifts from Randall Moon [49]). The pRenilla-CMV luciferase vector #E2261 (Promega; Madison, WI, USA) was used as an internal transfection control for all luciferase assay transfections, using a 1:10 Renilla luciferase to firefly luciferase ratio. Luciferase readings were taken 48 hours following transfection, using the Dual-Glo luciferase assay kit (Promega, #E2920) as per the manufacturer's instructions. All luciferase assays were performed in triplicate and repeated a minimum of three times. Results represent the mean of three separate experiments.

### RNA Isolation and Real-Time PCR Analysis

The QIAGEN RNeasy mini kit (Qiagen; Valenica, CA, USA) was used to isolate and purify RNA, as per the manufacturer's directions. The NanoDrop ND 1000 Spectrophotometer was used to measure RNA concentration. The purified and quantified RNA was used for cDNA preparation using the Oligo (dT) 12-18 Primer #18418-012 (Life Technologies) and Super Script II RNase reverse transcriptase (Invitrogen, Life Technologies). For Real-time PCR analysis, the Light Cycler 480 Real-Time PCR System (Roche; Indianapolis, IN, USA) was used. A 2x SYBR® green mix (Life Technologies) along with the appropriate primers and nuclease free water was used in a master mix (9 μl), which was added to 20 ng of cDNA per reaction. Real time PCR primers are enumerated in Supplementary Table B.

### Western Blot Analysis

Western blot analysis was performed as previously described [50]. Lysates were collected at 70-80% confluency. For the γ-secretase treatment, cells were seeded at 50-60% confluency, i.e., 4 × 10^5^ cells per well in a 6 well plate 24 hours prior to treatment. The γ-secretase inhibitor DBZ (EMD Milipore, CAS 209984-56-5; # 565789) was diluted in DMSO and treatment concentration was 500nM. Control cells were treated with an equal volume of DMSO. Following a freeze-thaw cycle, lysates were syringe-passed through a 21^5/8^ gauge needle. The Bio-Rad protein assay kit (Hercules, CA, USA) was used to quantify proteins in the lysates. For western blot analysis, a 10% SDS-PAGE gel was loaded with 20 – 40 μg of whole cell lysates for all proteins described, with the exception of MUC4, which was resolved on a 2% agarose gel due to its high molecular weight. The proteins were transferred onto a polyvinylidene difluoride membrane (Millipore; Billerica, MA, USA) and probed with primary antibodies overnight at 4°C. Details of the antibody suppliers and dilutions used are given in Supplementary Table A. On the following day, membranes were washed with PBST (4 × 10 min), and were incubated with the appropriate secondary antibodies (1:2000 dilution in PBS with 5% milk) for 45min-1 hour at room temperature. Subsequently, membranes were washed with PBST (4 × 10 min) again, and the ECL chemiluminescence kit (GE Healthcare Bio-Sciences, Pittsburgh, PA, USA) was used to visualize protein bands.

### Constructs

The generation of the 4ACAT stabilized β-catenin construct, where the residues S33, S37, T41 and S45 were mutated to Alanine and the *MUC4* promoter construct has been described in a previous publication [35]. For the Hath1 overexpression construct, the Hath1 cDNA was amplified from the HCT116 cell line using the following primers: HindIII-Hath1-FP: 5′-CATAAATAAGCTTTC CCGCCTGCTGCATGCAGAAG-3′and BamHI-Hath1- RP:5′-CTACAATGGATCCCTAACTTGCCTCATCCG AGTCAC-3′. The amplified cDNA was then ligated into the PCR2.1 vector using TOPO-TA cloning kit (Thermo Fisher Scientific #K451020). Following a restriction digestion with HindIII and BamH1, the released insert cloned into the HindIII and BamHI (New England Biolabs, #R0104Sand #R0136S) digested p3XFLAG- CMV-10 vector (Sigma-Aldrich, # E7658) and sequenced at the UNMC sequencing core facility.

### Promoter Analysis

The *MUC4* promoter was analyzed with the MatInspector software (Genomatix GmbH; Munich, Bavaria, Germany). Putative transcription factor binding sites with a matrix similarity score of > 0.85 were selected.

### Statistical Analysis

Data was analyzed using two-tailed T test with unequal variance using Microsoft® Office software. A p value of less than or equal to 0.05 was considered significant.

## SUPPLEMENTARY FIGURES AND TABLES


