# Supplementary material for: MUC4 is negatively regulated through the Wnt/β-catenin pathway via the Notch effector Hath1 in colorectal cancer
Source: Genes Cancer. 2016 May;7(5-6):154–68. doi: 10.18632/genesandcancer.108 (PMC4979589; doi:10.18632/genesandcancer.108)
Supplement: Supplementary file 1 [file ganc-07-154-s001.pdf]

MUC4 is negatively regulated through the Wnt/β-catenin pathway via the Notch effector Hath1 in colorectal cancer – Pai et al

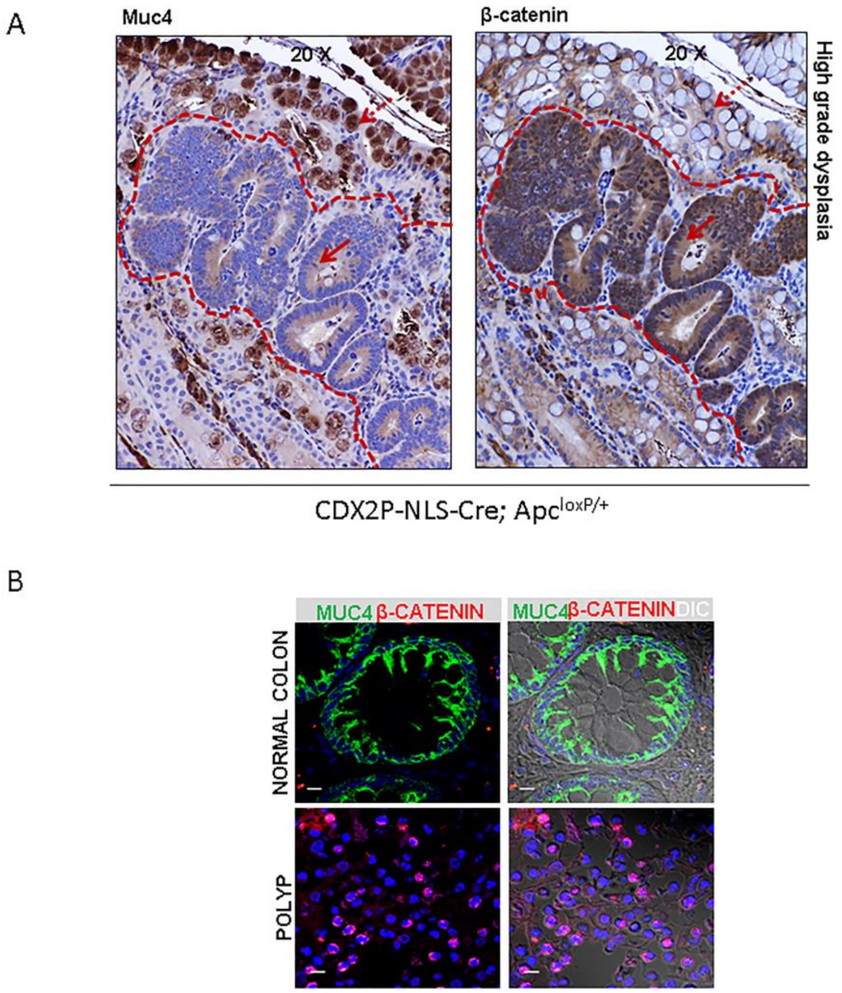

Supplementary Figure 1: (A) Immunohistochemical staining of mouse colon dysplasia. On the left, intense staining for Muc4 in goblet cells (dotted arrow) and weak, cytoplasmic staining in tumor cells (solid arrow) was observed. Dotted lines demarcate lesion. On the right, intense cytosolic/nuclear staining for β-catenin in the lesion (solid arrow) and weak staining in the adjacent normal areas (dotted arrow) was observed. Tissues were taken from colonic sections of

a mouse aged 14 weeks. **(B)** Confocal microscopy showed that MUC4 (green) was reduced in a polyp in comparison to the normal colon, concurrent with an increase in aberrant (cytosolic/nuclear) β-catenin (red).

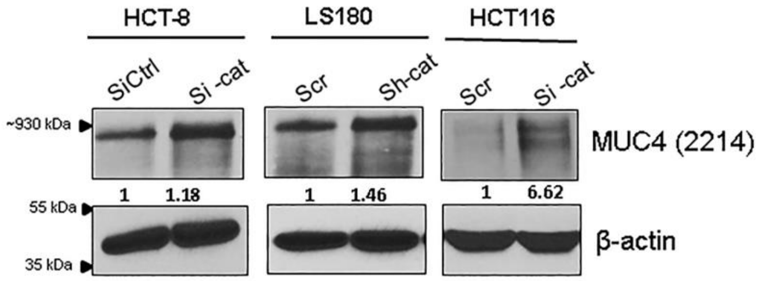

Supplementary Figure 2: In order to ensure that elevated MUC4 was not due to enhanced detection by the 8G7 antibody due to altered glycosylation, HCT-8, LS180 and HCT116 control and KD cells were probed with the 2214 MUC4 antibody, targeting the MUC4-α-N-Ter.

**Supplementary Table A**				
**Antibody**	**Supplier**	**Catalog no.**	**References**	**WB dilution**
β-catenin	Sigma-Aldrich	C2206	[51]	1:4000
β-catenin	BD Biosciences	610154	[52]	1:1000
MUC4(8G7)	Generated in our lab	Clone 8G7	[53]	1:1000
MUC4(2214)	Generated in our lab	Clone 2214	[24]	1:1000
β-actin	Sigma Aldrich	A5316	[54]	1:10,000
FLAG	Cell Signaling	8146	[55]	1:1000
Hes1	Santa Cruz. Gift; Dr. Punita Dhawan, UNMC	sc-25392	[56]	1:800
Mouse Muc4	Santa Cruz	sc-33654	[57]	-

Supplementary Table B: Real-time PCR/RT-PCR primers		
**Primer**	**Forward primer (5’-3’)**	**Reverse primer (5’-3’)**
β-catenin	CCTGGTGAAAATGCTTGGTTCAC	GAAGGCAGTCTGTCGTAATAGCC
MUC4	GACTTGGAGCTCTTTGAGAATGG	TGCAATGGCAGACCACAGTCC
β-actin	TGGACATCCGCAAAGACCTG	CCGATCCACACGGAGTACTT
Hath1	CGAGAGAGCATCCCGTCTAC	TCCGGGGAATGTAGCAAATA
$Apc^{fl/fl}$	GAGAAACCCTGTCTCGAAAAAA	AGTGCTGTTTCTATGAGTCAAC
Cdx2-Cre	GCGGTCTGGCAGTAAAAACTATC	GTGAAACAGCATTGCTGTCACTT
